# Silent Players, Loud Impact: The Influence of lncRNAs on Melanoma Progression

**DOI:** 10.3390/cancers17244033

**Published:** 2025-12-18

**Authors:** Kajetan Kiełbowski, Maciej Ćmil, Aleksandra Dach, Aleksandra Cole, Oliwia Jerzyńska, Estera Bakinowska, Paulina Plewa, Andrzej Pawlik

**Affiliations:** Department of Physiology, Pomeranian Medical University, 70-111 Szczecin, Poland; kajetan.kielbowski@onet.pl (K.K.); ola@aleksandracole.co.uk (A.C.); jerzynska.oliwia@wp.pl (O.J.); ebakinowska@op.pl (E.B.); paulina.plewa@op.pl (P.P.)

**Keywords:** oncology, melanoma, long non-coding RNA, transcription, post-transcriptional regulation

## Abstract

Non-coding RNA (ncRNA) encompasses a large family of molecules that are crucial regulators of gene expression. This family includes microRNA, piwi-interacting RNA, and long non-coding RNA (lncRNA); each class is associated with different mechanisms of action that influence gene expression. Based on the available evidence, these molecules have important roles in physiological and pathological processes. For example, ncRNAs are strongly implicated in oncogenesis by mediating the expression of tumour suppressors and oncogenes. This review comprehensively describes the latest findings regarding the roles of lncRNAs in the pathophysiology of melanoma. Key aspects of melanoma biology and various mechanisms regulated by lncRNAs are discussed. Furthermore, future areas exploring potential biomarkers and therapeutic targets are presented.

## Simple Summary

It is widely known that non-coding RNAs (ncRNAs) have a notable involvement in physiological and pathological processes. One of the most studied areas regarding ncRNAs is oncogenesis. By modulating gene expression, ncRNAs influence the activity of tumour suppressors and oncogenes. This review aims to comprehensively analyse and discuss the latest findings regarding the role of long ncRNAs (lncRNAs) in the pathogenesis of melanoma.

## 1. Introduction

Melanoma is a malignant neoplasm that can develop in the skin, mucous membrane, and uvea. It is the 17th most common type of malignancy and 22nd most frequent cause of cancer-related mortality [1]. Melanoma originates from cells that produce melanin, known as melanocytes. The disease has a multifactorial pathogenesis, with major involvement of germline mutations and ultraviolet radiation (UVR), which drives the occurrence of oncogenic mutations within the *BRAF* or *NRAS* genes, among others [2]. In the field of oncology, melanoma is an excellent example of continuous progress in treatment. The use of immunotherapy or kinase inhibitors in the treatment of patients with melanoma is now part of daily clinical practice [3]. The impressive effects of these treatments have been achieved due to comprehensive investigations of melanoma pathophysiology and progress in pharmacology. Nevertheless, researchers continue to examine the background mechanisms driving the progression of cancer to identify novel therapeutic targets or adequate biomarkers.

Non-coding RNAs (ncRNAs) encompass several subgroups that play a huge role in regulating gene transcription. Although they have been known for years, research interest in ncRNAs has increased substantially after Victor Ambros and Gary Ruvkun were awarded with the Nobel Prize for the discovery of microRNA (miRNA) [4]. Classically, miRNA binds to target messenger RNA (mRNA) to suppress its translation. Long non-coding RNA (lncRNA) represents another group of ncRNA that can interact with miRNAs to release their suppression of translation. Based on the consensus statement by Mattick et al. [5], lncRNAs contain more than 500 nucleotides. Hundreds of thousands of human lncRNAs have been identified. They mediate numerous physiological processes such as cellular proliferation, differentiation, or apoptosis, and they have been highly studied in the field of oncology [6]. It is known that dysregulated expression of ncRNAs can strongly contribute to the development of cancer. This review aims to discuss the latest findings regarding the role of lncRNAs in the pathophysiology of melanoma.

## 2. Molecular Mechanisms Regulated by Long Non-Coding RNA

### 2.1. Transcriptional Regulation

#### 2.1.1. Long Non-Coding RNA and Chromatin-Modifying Complexes

Polycomb repressive complex 2 (PRC2) is a multiprotein complex responsible for the epigenetic repression of gene transcription. Its main function is the trimethylation of lysine 27 on histone H3 (H3K27me3), catalysed by enhancer of zeste homolog 2 (EZH2). This histone modification leads to chromatin condensation, which consequently results in the silencing of target gene expression [7]. PRC2 itself exhibits limited ability to recognise specific DNA sequences. However, lncRNAs can bind to PRC2, particularly to EZH2. These interactions help guide the complex to specific genomic loci, modifying its target specificity and enabling selective transcriptional repression [8]. In melanoma, lncRNAs regulate EZH2 activity, thereby influencing tumour progression. Several lncRNAs act as oncogenes by recruiting EZH2 to repress tumour suppressors. For example, FOXC2-AS1 promotes melanoma cell proliferation by silencing the tumour suppressor gene cyclin-dependent kinase inhibitor 2B *CDKN2B* (which encodes p15INK4b) through an EZH2-dependent recruitment mechanism [9]. However, lncRNAs with tumour-suppressive and prognostically favourable functions have also been identified. LINC-PINT binds to EZH2 and represses oncogene expression, including *CDK1*, *AURKA*, and *CCNA2*. As a result, melanoma cell proliferation and metastasis are reduced [10].

#### 2.1.2. Long Non-Coding RNA Interaction with Transcription Factors and Co-Activators

Another mechanism by which lncRNAs regulate transcription is through their interactions with transcription factors. E2F1 plays a critical role in cell cycle regulation and has been established as an oncogene in melanoma cells [11]. Its correlation with the lncRNA SLNCR1, whose elevated expression is associated with poor prognosis, has been investigated [12]. Researchers demonstrated that the formation of the SLNCR1–E2F1 complex guides E2F1 to activate genes, promoting tumour progression. Importantly, disruption of this complex, without altering the levels of SLNCR1 or E2F1, significantly reduces the invasive capacity of melanoma cells. These findings suggest that targeting the RNA–protein interaction itself may represent a potential therapeutic strategy [13].

#### 2.1.3. Enhancer-Associated lncRNAs and Enhancer RNAs (eRNAs)

eRNAs are a class of ncRNA transcribed from enhancer regions. Increasing evidence indicates that eRNAs can actively modulate transcription, thereby influencing tumour development and progression [14]. A recent study by Zhao et al. [15] reported the involvement of two eRNAs, LINC00689 and ELFN1-AS1, in uveal melanoma. High expression of LINC00689 is associated with a favourable prognosis and longer overall survival, whereas overexpression of ELFN1-AS1 correlated with a poorer prognosis, suggesting its potential oncogenic function. For LINC00689, co-regulated genes are mainly involved in antigen presentation, the immune response, and cell adhesion, indicating a potential role in tumour–immune interactions. In contrast, ELFN1-AS1-regulated genes are associated with melanogenesis and extracellular matrix organisation, potentially promoting increased cell migration and invasiveness. Gan et al. [16] integrated eRNA analyses with immune-related data, allowing them to construct a prognostic profile for melanoma that included AC009495.2, LINC02446, and LINC00189, among others. The expression levels of these eRNAs accurately distinguished patients depending on their survival outcomes. Importantly, after adjusting for other clinical variables, the expression levels of these eRNAs remained significant independent prognostic indicators.

### 2.2. Post-Transcriptional Regulation

#### 2.2.1. Competing Endogenous RNA (ceRNA) Networks: The lncRNA–miRNA–mRNA Axis

The ceRNA hypothesis describes how lncRNAs can bind miRNAs through complementary base-pairing at microRNA response elements (MREs), preventing miRNAs from silencing their target mRNAs. Under normal conditions, many miRNAs act as tumour suppressors by binding oncogenic transcripts, promoting their degradation or translational repression. When lncRNAs act as molecular sponges, they sequester these miRNAs, reducing miRNA-mediated silencing and allowing oncogenic mRNAs to be expressed. In melanoma, metastasis-associated lung adenocarcinoma transcript 1 (MALAT1) serves as an illustrative example: it sponges miR-22 and contributes to sustained mitogen-activated protein kinase (MAPK)-pathway signalling [17,18]. Its detailed roles in melanoma are discussed in Section 4. This mechanism highlights how ceRNA networks regulate post-transcriptional gene expression.

#### 2.2.2. Splicing Modulation of Long Non-Coding RNA

The influence of lncRNAs on splicing modulation is well documented: they can interact with specific splicing factors and modulate their function; form RNA–RNA duplex structures with pre-mRNA, affecting splicing outcomes; and even influence chromatin organisation, indirectly shaping the splicing of target genes [19]. The involvement of splicing factors in regulating lncRNA expression has been observed and linked to melanoma pathogenesis. Zhang et al. [20] demonstrated that the splicing factor polypyrimidine tract binding protein 1 (PTBP1) promotes the splicing of the precursor LHFPL3-AS1, leading to the generation of the LHFPL3-AS1-long isoform. LHFPL3-AS1-long then binds miR-181a-5p, preventing its interaction with Bcl-2 mRNA and thereby blocking its degradation. As a result, Bcl-2, an anti-apoptotic protein, is produced, supporting the survival of melanoma stem cells, and promoting tumour maintenance. Similarly, silencing PTBP1 decreases Bcl-2 and LHFPL3-AS1-long expression and reduces tumour size.

#### 2.2.3. Long Non-Coding RNA and Translational Control

As described above, LHFPL3-AS1-long blocks miRNA binding to specific mRNAs, preventing their degradation. This is an example of a lncRNA influencing mRNA stability. Another important regulatory mechanism is the control of translation, which determines when and how much of the mRNA present in the cell is converted into protein. This allows the cell to adapt to changing conditions without requiring changes at the transcriptional level. In melanoma, translation plays a key role in regulating cell phenotype, determining the switch between proliferative and invasive states. For example, the lncRNA terminal differentiation-induced non-coding RNA (TINCR) inhibits the invasive phenotype of melanoma, partly through the regulation of activating transcription factor 4 (ATF4), a stress-response factor that can regulate protein translation, enhancing invasion. At the beginning of its mRNA, there are upstream open reading frames (uORFs) 1 and 2–short sequences located before the main start codon that act as a ‘brake’ on ATF4 synthesis. Ribosomes first translate uORF1, then uORF2, which consequently results in low ATF4 production. In response to stress, eukaryotic initiation factor 2 α subunit (eIF2α) becomes phosphorylated, which slows the initiation of translation for most mRNAs but allows ribosomes to more easily ‘bypass’ uORF2, increasing ATF4 translation. TINCR knockdown increases ATF4 translation despite the lack of change in uORF1/2 or eIF2α phosphorylation, indicating an alternative mechanism of ATF4 translational regulation. Moreover, TINCR knockdown reduces the phosphorylation of elongation factor 2 (EEF2), which normally slows translation elongation, limiting ribosome movement along mRNA. As a result, global elongation accelerates and ATF4 translation is enhanced, favouring the invasive phenotype [21].

#### 2.2.4. Interaction with RNA-Binding Proteins

Testis-associated highly conserved oncogenic long non-coding RNA (THOR) is also involved in post-transcriptional regulation. It forms a complex with insulin-like growth factor 2 mRNA-binding protein 1 (IGF2BP1), an mRNA-binding protein that plays a key role in post-transcriptional gene regulation. IGF2BP1 is expressed in foetal tissues and in more than 16 types of cancers, while its expression in normal adult tissues is low [22]. Binding to THOR enhances the ability of IGF2BP1 to interact with its target transcripts and protect them from degradation. As a result, high levels of oncogenic mRNAs, such as insulin-like growth factor 2 (IGF2), c-MYC, and CD44, are maintained. In in vivo models, THOR overexpression promotes the initiation and progression of melanoma, whereas its absence is associated with the inhibition of tumourigenesis [23].

## 3. Key lncRNAs in Melanoma Pathogenesis

### 3.1. Oncogenic lncRNAs

Melanoma is associated with significant changes in lncRNA expression. For example, Ma et al. [24] identified over 1600 differently expressed lncRNAs in tissues of patients with melanoma compared with samples of nevus tissue. Considering the wide array of functions performed by these molecules, they markedly affect cellular behaviour. In the area of oncology, the influence on intracellular signalling pathways is a crucial mechanism leading to abnormal proliferation, resistance to apoptosis, and abnormal differentiation, classic markers of oncogenesis. Different signalling pathways are frequently interconnected with each other, creating a network that can be difficult to target precisely. ncRNAs regulate these relationships and signalling pathways.

CD27-AS1-208 is an antisense lncRNA that is upregulated in primary and metastatic melanoma tissues. Furthermore, its expression correlates positively with Ki67, suggesting an important role in driving the aggressiveness of the malignancy, as confirmed in experiments evaluating migration and proliferation, which are disrupted in cells with CD27-AS1-208 knockout. Mechanistically, CD27-AS1-208 interacts with signal transducer and activator of transcription 3 (STAT3), and its knockdown reduces the phosphorylation of STAT3. Consequently, downstream elements of STAT3 are also downregulated [24]. Given that STAT3 is one of the crucial pathways involved in melanoma, agents that suppress melanoma progression can do so by inhibiting STAT3 activity [25,26]. Furthermore, STAT3 is considered important in the processes regulating melanoma metastasis. Nevertheless, STAT3 can also suppress the growth of cancer cells depending on the cellular context or stage of disease [27]. Therefore, STAT3 can promote metastasis but may suppress tumor growth depending on tumor stage/environment.

**Figure 1:**
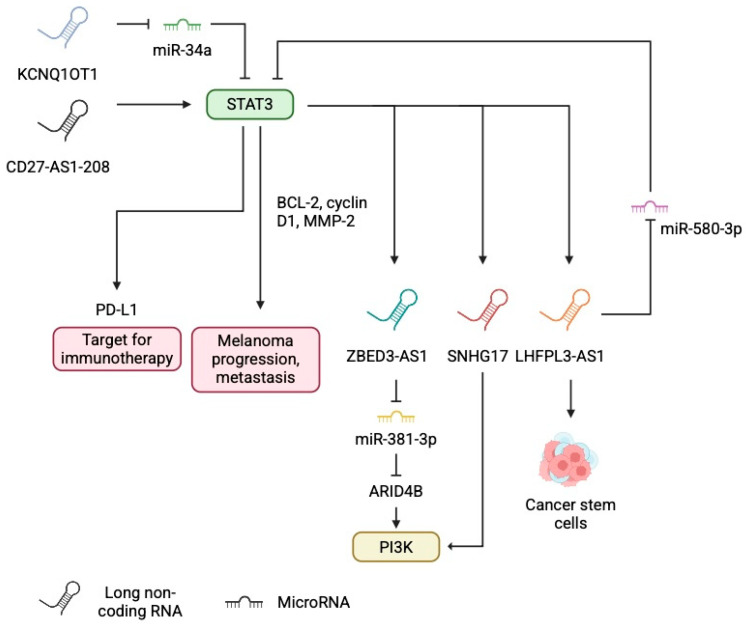
Impact of long non-coding RNA on signalling pathways, focusing on STAT3. STAT3 is an element of the key signalling pathway that orchestrates major cellular functions, including immune checkpoint expression. Therefore, we suggest that modulation of STAT3 by lncRNA can influence the immunotherapy response. CD27-AS1-208 and KCNQ1OT1 are lncRNAs that were recently demonstrated as upstream regulators of STAT3. At the same time, transcription factors influence the expression of several lncRNAs that then modulate melanoma functionality and are involved in signalling loop with STAT3 itself or regulate other signalling pathways. Created in BioRender. Physiology, D. (2026) https://BioRender.com/5gozbqo.

STAT3 is involved in a much denser network of interactions with ncRNAs. As a transcription factor, it also affects the expression of other lncRNAs. For example, Peng et al. [28] demonstrated that STAT3 regulates the expression of LHFPL23-AS1, the expression of which is increased in melanoma tissues compared to non-tumour samples. This molecule is involved in a feedback loop; it acts as a sponge for miR-580-3p, further increasing the expression of STAT3 in a post-transcriptional mechanism. Similarly, the KCNQ1OT1–miR-34a axis regulates STAT3 expression in melanoma. High KCNQ1OT1 expression is associated with poorer survival and increased levels of STAT3 [29]. Importantly, STAT3 is an upstream regulator of PD-L1 [30,31,32], an immune checkpoint protein that suppresses the anticancer properties of immune cells. Inhibiting the STAT–PD-L1 axis is an anticancer mechanism in melanoma [33]. As melanoma is one of the malignancies for which immunotherapy plays a crucial role, understanding the regulators of PD-1/PD-L1 expression and activity is very relevant. KCNQ1OT1 regulates the expression of PD-L1 [29]. Another lncRNA upregulated by STAT3 is ZBED3-AS1, the expression of which is increased in melanoma and is associated with worse survival. ZBED3-AS1 links the STAT3 pathway with the PI3K–Akt signalling cascade, which is involved in oncogenesis, including the biology of melanoma [34,35]. ZBED3-AS1 sequesters miR-381-3p, thus upregulating AT-rich interaction domain 4B (ARID4B), which can activate the PI3K–Akt pathway [36]. High expression of ARID4B has been associated with other types of neoplasms. For example, researchers identified it as a biomarker of disease-free survival and overall survival in hepatocellular carcinoma [37]. There is a similar mechanism regarding the lncRNA SNHG17: it is upregulated in melanoma tissues, and its high expression correlates with the presence of lymph node metastasis, tumour stage, and poor prognosis. Similarly to ZBED3-AS1, expression of SNHG17 is regulated by STAT3, and it can activate the PI3K–Akt signalling pathway (Figure 1) [38].

Both activation of oncogenes and downregulation of tumour suppressors within the PI3K–Akt signalling cascade contribute to tumourigenesis. Targeting phosphatidylinositol-4,5-bisphosphate 3-kinase catalytic subunit alpha (PIK3CA) mutations with alpelisib in breast cancer and mutations of the tumour suppressor PTEN are typical examples. As demonstrated above, the pathway is interconnected with regulators of the STAT3 cascade. Nevertheless, other molecules mediate the activity of the PI3K–Akt signalling. X-inactive specific transcript (XIST) is a lncRNA that is upregulated in melanoma cells. It acts as a molecular sponge for miR-21, inhibiting the expression of phosphoinositide-3-kinase, regulatory subunit 1 (PI3KRI) and AKT, and promoting Bcl-2 and Bax expression [39]. The lncRNA myocardial infarction-associated transcript (MIAT) also leads to activation of the PI3K–AKT signalling pathway and is implicated in the progression of the disease. Moreover, it stimulates expression of c-MYC and cyclin D1 [40]. LINC00511 promotes phosphorylation of PI3K and Akt but does not regulate their expression. Mechanistically, LINC00511 is regulated at the transcriptional level by the yin yang 1 (YY1) transcription factor. Then, the molecule acts through the miR-150-5p–a disintegrin and metalloproteinase 19 (ADAM19) axis to ultimately upregulate the protein expression that activates the PI3K signalling pathway [41].

Melanoma cells are also characterised by overexpression of the lncRNA sprouty4-intron 1 (SPRY4-IT1) and low expression of miR-22-3p. Upregulation of miR-22-3p inactivates the MAPK signalling pathway by inhibiting the phosphorylation of p38MAPK, mitogen-activated protein kinase-activated protein kinase (MAPKAPK), and heat-shock protein 27 (Hsp27), suppressing the proliferation of melanoma cells. Therefore, low expression of SPRY4-IT1 inhibits the proliferation, invasion, migration, and epithelial–mesenchymal transition (EMT) of neoplastic cells [42]. Mitogen-activated protein kinase kinase (MEK), part of the MAPK pathway, phosphorylates and activates ERK. BRAF-activated non-protein coding RNA (BANCR) is an lncRNA that was first identified in a study analysing gene expression in melanocytes in the context of BRAF mutations. The BRAFV600E mutation leads to constitutive activation of BRAF kinase, resulting in persistent stimulation of the MAPK–extracellular signal-regulated protein kinase (ERK) pathway and, consequently, increased expression of the *BANCR* gene. BANCR subsequently contributes to tumourigenesis by regulating the activity of key cellular signalling pathways, particularly MAPK–ERK, thereby creating a positive feedback loop and influencing gene expression through post-transcriptional mechanisms. Elevated BANCR expression has been observed in a variety of cancers, including melanoma [43]. In a study on hepatocellular carcinoma, researchers linked BANCR to MEK: reducing BANCR expression inactivates the MEK pathway [44]. Flockhart RJ et al. [45] used small hairpin RNA (shRNA) to reduce BANCR expression in melanoma cells to less than 25% of normal levels. They reported changes in the expression of 88 genes, some of which are associated with cell motility and chemotaxis. Cell survival, however, is not affected, indicating that BANCR is not essential for cell viability. Additional studies showed that the level of the chemokine C-X-C motif chemokine 11 (CXCL11), which is critical for cell migration, decreases after BANCR silencing. Restoration of CXCL11 expression rescues the migratory capacity of the cells, suggesting that BANCR indirectly regulates melanoma cell migration through CXCL11. In a subsequent study using shRNA, researchers investigated the effect of BANCR on melanoma cell proliferation [43]. They found that knockdown of BANCR decreases proliferation across three independent melanoma cell lines. This effect is enhanced when the cells are treated with inhibitors of ERK1/2 and c-Jun N-terminal kinase (JNK), which are components of the MAPK pathway. This combined treatment markedly reduces proliferation, whereas artificial overexpression of BANCR in these cells partially rescued proliferation. It is well established that both ERK and JNK are involved in cell migration [46,47], regulating cytoskeletal remodelling, cell adhesion, and the activity of genes responsible for motility and invasion.

**Figure 2:**
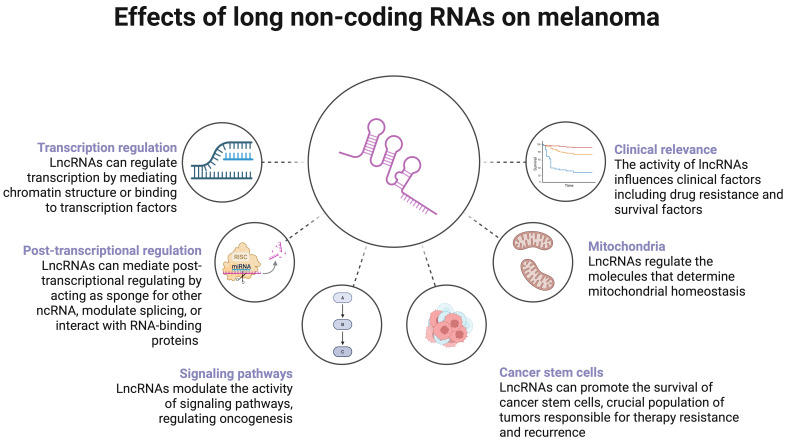
Effects of long non-coding RNA molecules on melanoma behaviour and their clinical relevance. Created in BioRender. Physiology, D. (2026) https://BioRender.com/kk0goju.

Single lncRNAs are usually involved in a variety of different interactions and therefore exert several functions. For example, LHFPL3-AS1 is involved in the behaviour of cancer stem cells, a population of cells within the tumour with stem-like properties that are capable of inducing tumour regrowth [48]. Zhang et al. [20] demonstrated that silencing LHFPL3-AS1 inhibits proliferation of melanoma stem cells. Mechanistically, this lncRNA suppresses degradation of Bcl-2 by interacting with miR-181. Importantly, while ncRNAs are frequently investigated in various malignancies, the precise effects in melanoma still need to be verified. LHFPL3-AS1 has been examined in head and neck cancer; it is associated with chemo- and radiotherapy resistance [49,50]. Much more evidence exists regarding the potential roles of the lncRNA KCNQ1OT1. Researchers have demonstrated that this molecule promotes the growth of bladder cancer [51], ovarian cancer [52], and colorectal cancer [53], among others. Interestingly, a study investigating the involvement of KCNQ1OT1 in ovarian cancer demonstrated an important feature of this lncRNA. It can also regulate gene expression by modulating DNA methylation and is thus involved in transcriptional regulation. Specifically, KCNQ1OT1 can recruit DNA methyltransferase to influence the progression of ovarian cancer cells [54]. Examples of lncRNA involvement in melanoma tumorigenesis are presented on Figure 2.

### 3.2. Tumour-Suppressor lncRNAs

*DIRC3* was discovered in 2003 as an unknown gene in a family with hereditary renal cell carcinoma. It was proposed as a tumour suppressor in 2019 in a landmark study by Coe et al. [55]. DIRC3 acts in the nucleus and has been shown to activate expression of the neighbouring insulin-like growth factor binding protein 5 (*IGFBP5*) gene, which shapes tumour suppression by modulating the insulin-like growth factor 1 receptor (IGF1R) pathway. The role of DIRC3 in tumour suppression in melanoma is based on the modulation of transcriptional networks governed by MITF and SOX10. MITF upregulation is frequent in melanoma, especially in the metastatic phase. MITF and SOX10 are said to bind concurrently to chromatin and control key gene regulation. DIRC3 modifies the local chromatin conformation, reducing SOX10 binding to enhancer elements within its own locus, thereby enabling transcriptional activation of IGFBP5. The authors identified 245 candidate lncRNAs associated with melanoma whose loci are jointly bound by MITF and SOX10, suggesting that DIRC3 exerts broad tumour-suppressive effects through IGFBP5-dependent mechanisms. Importantly, downregulation of DIRC3 is strongly associated with poor prognosis and decreased overall survival in patients with melanoma. Loss of DIRC3 through CRISPR interference markedly enhances anchorage-independent growth in a number of melanoma cell lines, which indicates metastatic potential. IGFBP5 reduction produces comparable results, confirming that DIRC3 mediates tumour suppression via IGFBP5 activation. Clinical data from The Cancer Genome Atlas (TCGA) further strengthens these findings, showing low DIRC3 expression is strongly associated with reduced patient survival. Interestingly, the data point to DIRC3 functioning as a feedback modulator within the MITF–SOX10 network. Specifically, this network suppresses DIRC3 transcription, which in turn regulates chromatin accessibility to limit SOX10 and maintain expression of tumour-suppressive genes such as IGFBP5. Therapeutic upregulation of DIRC3 may therefore represent a promising strategy for melanoma [55].

Growth arrest-specific transcript 5 (GAS5) is an ncRNA that is host for several intronic small nucleolar RNAs (snoRNA) that methylate ribosomal RNA (rRNA). snoRNAs can exhibit either tumour-suppressive or pro-tumourigenic effects, depending on the snoRNA type and the context. GAS5 levels are frequently downregulated in neoplastic tissues, which might be due to altered transcriptional and mammalian target of rapamycin (mTOR)-dependent post-transcriptional control [56]. Clinical samples of melanoma have consistently shown that GAS5 is downregulated in advanced disease, correlating with greater tumour thickness, ulceration, and nodal metastasis. Functionally, knocking down GAS5 in melanoma cells accelerates G1/S progression through increases in cyclin D1, CDK4, and p27; boosts viability; and blunts apoptosis via increased expression of Bcl-2. Conversely, overexpression of GAS5 has the opposite effect. Mechanistically, GAS5 helps restrain oxidative metabolism. When knocked down, there are elevated levels of NADPH oxidase 4 (NOX4) and NADPH oxidase activity, which results in a shift in the redox state with an increase in NADP+, oxidised glutathione, and NOX activity. GAS5 was also found to physically interact with glucose-6-phosphate dehydrogenase (G6PD), linking GAS5 to pentose-phosphate-driven redox control [57]. Furthermore, GAS5 suppresses EZH2 by recruiting the transcription factor E2F4 to its promoter. This results in reduced H3K27 trimethylation and leads to upregulation of the tumour suppressor CDKN1C. GAS5 reduction also correlates with poorer patient survival [58]. The molecule has been demonstrated to interact with numerous miRNAs to promote tumour suppression. Thus, many signalling pathways are modulated by its regulatory network across different cancers, including Wnt–β-catenin [59], PI3K–AKT [60], AKT–mTOR [61], Hedgehog [62], and Hippo [63].

A recent study investigated GAS5 methylation levels in melanoma to provide therapeutic targets. DNA-methylation-induced gene silencing plays a crucial role in tumour initiation and progression, while demethylating agents can reactivate these silenced genes and restore their antitumour biological functions. The results revealed that the downregulation of GAS5 expression in melanoma may be correlated with modification of methylation. In melanoma tissues and cells, the CpG islands in the promoter region of GAS5 are hypermethylated, while there is no methylation in normal tissues. The authors reported that treatment of melanoma cells with decitabine, a demethylating agent, reverses GAS5 methylation and reduces cancer invasion [64].

LncRNA maternally expressed gene 3 (MEG3), which is present in the nucleus and cytoplasm [65], has a tumour-suppressive function across cancers, including colorectal [66], cervical [67], lung [68], gastric [69], ovarian [70], glioma [71], breast [72], liver [73], and osteosarcoma [74], among others. Interestingly, there are many different mechanisms reported in suppressing tumour cell proliferation. In gastric carcinoma, MEG3 seems to regulate the expression of anti-apoptotic Bcl-2 and Bcl-2-like protein 2, thereby inhibiting caspase-9 and caspase-3. However, this has not yet been verified in a melanoma model [75]. MEG3 is downregulated in melanoma. Its overexpression was associated with suppressed tumour growth in vitro and in vivo. Mechanistically, the identified axis of MEG3 was miR-208/SOX4, which was also proven to play a role in cancer stem cell functionality [76].

A recent study examined the association between the lncRNA POU3F3 and MEG3 in tissue samples from 60 patients with melanoma. The results confirmed the downregulation of MEG3 and the inverse correlation between POU3F3 and MEG3. Overexpression of POU3F3 is associated with increased melanoma cell proliferation, whereas overexpression of MEG3 induces the opposite effect. POU3F3 negatively influences MEG3 levels in melanoma cells, while overexpression of MEG3 does not affect POU3F3 levels, suggesting interactions between POU3F3 and other factors. Additionally, the expression of POU3F3 and MEG3 correlate in tumour tissue but not in normal tissue, pointing towards some undiscovered pathological mediators between POU3F3 and MEG3 [77].

In a recent study on the involvement of MEG3 in the regulation of melanoma cell behaviour, researchers found that MEG3 overexpression does not significantly alter pathways involving ERK, STAT, and PI3K. Instead, the authors demonstrated a link between MEG3 and hepatocyte growth factor (HGF). In normal tissues, HGF and mesenchymal–epithelial transition (MET) factor are key mediators of connective tissue signalling. However, in cancer, HGF–c-met signalling drives cell proliferation, survival, migration, invasion, and metastasis. This pathway is activated when secreted pro-HGF is cleaved by serine proteases into active HGF, which then binds and activates MET. Moreover, MEG3 is related to c-met. The HGF–c-met pathway operates in an autocrine manner in melanoma and is typically regulated in an inhibitory way by MEG3 [78]. Amplification of MET has been associated with worse survival in patients treated with immunotherapy [79]. MEG3 could serve as a therapeutic target to counteract resistance to HGF–c-met inhibitors through regulation of c-met signalling [78].

Wu et al. [80] revealed that MEG3 regulates melanoma progression through the miR-21–E-cadherin signalling pathway. First, the authors confirmed that melanoma tissue has lower MEG3 suppression relative to the surrounding area, and this expression corresponds to the survival rate. Second, E-cadherin levels are clearly decreased compared with non-tumour tissue. Moreover, MEG3 and E-cadherin corelate positively, and overexpression of E-cadherin decreases the migratory capacity of cells compared with their respective controls. The link molecule between MEG3 and E-cadherin is miR-21. Consistently, miR-21 has been shown to be overexpressed in primary cutaneous melanoma. The authors suggested that MEG3 attenuates levels of miR-21, which results in downstream target E-cadherin gene upregulation.

The level of MEG3 is also influenced by cytoglobin (CYGB), a hexacoordinated globin expressed in various tissues. This protein is highly abundant in melanocytes but frequently reduced during melanoma development. In cell lines with CYGB knockdown, MEG3 is dramatically downregulated, suggesting that CYGB influences MEG3 expression [81]. In in vitro and in vivo murine melanoma models, researchers reported increased MEG3 expression after treatment with gambogenic acid, leading to inhibition of EMT. Conversely, knockdown of MEG3 enhances melanoma cell proliferation, migration, and invasion, confirming its inhibitory role [82].

**Figure 3:**
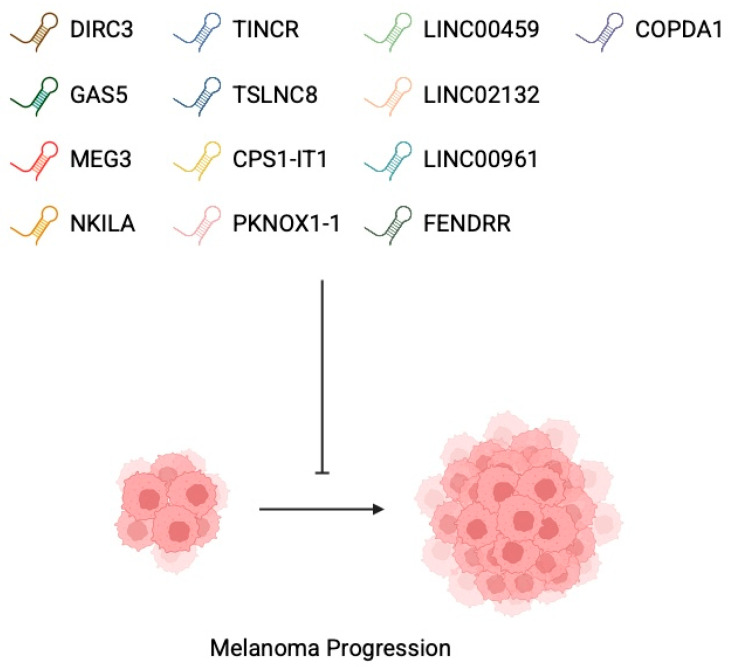
Similarly to oncogenic long non-coding RNAs, numerous molecules were also found to suppress the progression of the disease Created in BioRender. Physiology, D. (2026) https://BioRender.com/jxn63ob.

Several other lncRNAs were identified as melanoma suppressors. NF-κB interacting long non-coding RNA NKILA blocks IκB phosphorylation and modulates the NF-κB signalling pathway. NF-κB is a major inflammatory mediator that is involved in inflammatory and malignant diseases. It has already been demonstrated as a therapeutic target in melanoma [83,84]. NKILA reduced melanoma cell proliferation through targeting NF-κB [85]. LncRNA CPS1-IT1 suppresses development of melanoma metastasis by targeting cysteine-rich protein 61, an extracellular matrix-associated signalling protein also demonstrated to take part in melanoma tumorigenesis [86,87]. LINC00961 [88], LINC00495 [89], FENDRR [90], TINCR [21], TSLNC8 [91], Lnc-PKNOX1-1 [92], LINC02132, and COPDA1 [93] represent other lncRNa molecules found to inhibit melanoma tumorigenesis (Figure 3).

## 4. Hallmarks of lncRNA Involvement in Melanoma

### 4.1. Cell Proliferation and Cell Cycle Control

BANCR inhibits the expression of miR-204, a suppressor of melanoma cell growth. Thus, targeting BANCR may prevent proliferation and migration of malignant melanoma [94]. Another lncRNA, nuclear paraspeckle assembly transcript 1 (NEAT1), promotes melanoma growth and invasion via the miR-495-3p–E2F3 axis, inhibiting miR-495-3p and inducing E2F3, which is known to promote proliferation [95,96,97]. SNH5 serves as a ceRNA for miR-26a-5p, which downregulates transient receptor potential, canonical 3 (TRPC3), an ion channel largely implicated in basic cellular functions, including proliferation. Knockdown of TRPC3 suppresses proliferation of melanoma cells, confirming the involvement of the abovementioned axis in melanoma growth [98]. TRPC3 also promotes proliferation in other cancers [99]. The SPRY4-IT1–miR-22-3p axis also regulates cell growth. As previously mentioned, SPRY4-IT1 regulates the MAPK pathway, thus significantly contributing to cell proliferation [42]. GAS6-AS2 promotes tumour growth by activating AXL–AKT–ERK signalling [100]. Upregulation of MALAT1 promotes cell proliferation by stimulating the expression of cyclins D1 and E1 and downregulating caspase-3 and caspase-9 to ultimately suppresses apoptosis [101]. FOXC2-AS1 stimulates the proliferation of melanoma cells via silencing p15, a tumour-suppressor gene that prevents cell cycle progression from the G1 phase to S phase through recruiting EZH2 [9]. The overexpression of lncRNA-ATB also leads to an increase in the number of melanoma cells. The lncRNA-ATB–miR-590-5p–Yes1-associated transcriptional regulator (YAP1) also stimulates the migration and invasion of melanoma cells [102]. BASP1-AS1 interacts with Y-box binding protein 1 (YBX1), recruiting it to the neurogenic locus notch homolog protein 3 (NOTCH3) promoter. As a result, the Notch pathway is activated, leading to the transcription of c-MYC, proliferating cell nuclear antigen (PCNA), and CDK4, which are involved in melanoma progression [103]. TTN-AS1 dysregulates the cell cycle by upregulating cyclin D1, CDK2, and CDK4, promoting cell progression to the G2 phase. Overexpression of TTN-AS1 reduces the rate of apoptosis by downregulating cleaved caspase-3, cleaved caspase-9, and Bax. Overexpression of FOXF1 adjacent non-coding developmental regulatory RNA (FENDRR) reduces cyclin E1, cyclin D1, CDK4, and CDK6 levels, which arrests melanoma cells in the G1 phase. It also influences the stability of c-MYC by interacting with RNA-binding proteins, leading to low expression of c-MYC mRNA. As a result, the growth of melanoma cells is suppressed [90].

**Figure 4:**
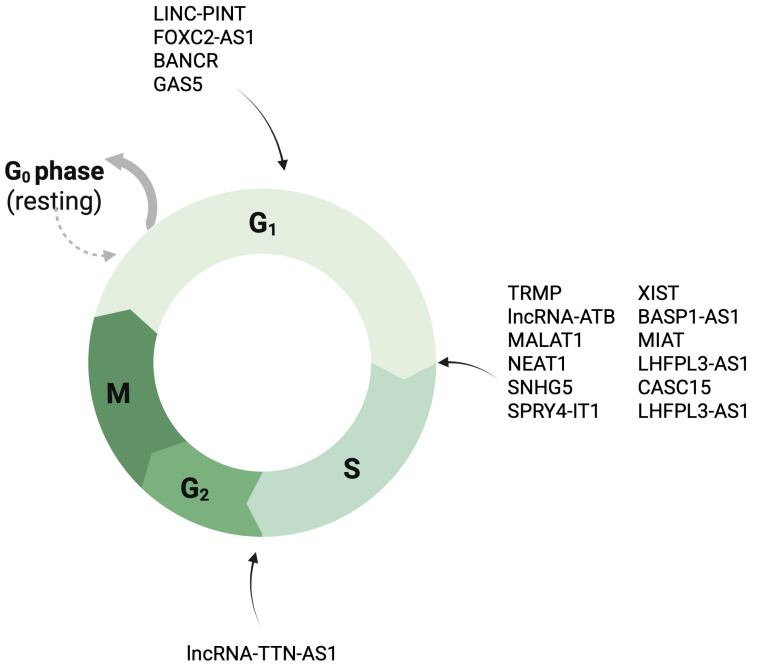
Overview of cell cycle and the involvement of long non-coding RNA in its progression across several cancers. Created in BioRender. Dach, A. (2026) https://BioRender.com/qowmko8.

TP53-regulated modulator of p27 (TRMP), a splice variant of the lncRNA RP11-369C8.1, is a vital effector of p53 activity that upregulates TRMP expression. This lncRNA presents a pro-survival role in cells by stimulating progression from the G1 phase to the S phase of the cell cycle. It also promotes metastasis by inhibiting p27. Knockdown of TRMP arrests cells in the G1 phase and thus inhibits proliferation [104]. A short splice variant (TRMP-S) inhibits p27 through a different pathway. While TRMP regulates p27 protein expression by competing with p27 mRNA for binding to PTBP1, TRMP-S stabilises ubiquitin-like with PHD and Ring Finger domains 1 (UHRF1), a p27 inhibitor, and suppresses p53 translation, leading to restrained p27 expression [104,105]. TRMP-S promotes the growth of cells and suppresses cellular senescence by inhibiting p53 mRNA translation. LINC-PINT is significantly downregulated in melanoma cells. When upregulated, LINC-PINT inhibits tumour growth and migration by recruiting EZH2, leading to H3K27 trimethylation and epigenetic silencing of target genes. As a result, melanoma cells are stopped in the G0/G1 phase of the cell cycle. Other potential targets of LINC-PINT are *CDK1*, *CCNA2*, *AURKA*, and *PCNA* [10]. Figure 4 briefly summarises the impact of lncRNAs on the cell cycle.

### 4.2. Programmed Cell Death

Apoptosis is a form of programmed cell death that is necessary to preserve homeostasis in the organism. It is controlled by the Bcl-2 family of proteins that consists of both pro-death and pro-survival proteins. LncRNAs regulate this process by affecting Bcl-2 expression. GAS5 promotes apoptosis in melanoma; GAS5 knockdown induces G1/S progression and inhibits apoptosis by upregulating Bcl‑2, whereas GAS5 overexpression decreases Bcl‑2 [57]. In contrast, LHFPL3-AS1 suppresses apoptosis by interacting with miR-181a-5p and thus inhibiting the degradation of Bcl-2 [20].

There is also programmed necrotic cell death, including an inflammatory form called pyroptosis, which has a dual effect on a tumour: it stimulates the anti-tumour pathway or triggers tumourigenesis. LncRNAs can initiate pyroptosis in tumour cells [106]. In melanoma, some pyroptosis-related lncRNAs may help predict prognosis in patients by indicating the melanoma cell infiltration status. Moreover, pyroptosis-related lncRNAs could support the exploration of potential therapeutic targets [107].

Ferroptosis is an iron-dependent form of programmed cell death that plays a pivotal role in tumour progression. AGAP2-AS1 downregulates ferroptosis by stimulating the expression of solute carrier family 7 member 11 (SLC7A11) through the IGF2BP2 pathway. Similarly, NEAT1 inhibits ferroptosis via increased expression of SLC7A11 [108].

### 4.3. EMT Transition

EMT is a multi-step process in which epithelial cells lose adherence and transform into mesenchymal cells, gaining motility and the ability to migrate to distant tissues in the organism as metastases [109]. It involves upregulation of N-cadherin and vimentin and deregulation of E-cadherin. This process is regulated by cell adhesion molecules that mediate binding between cells and the extracellular matrix. The Wnt–β-catenin pathway controls the expression of E-cadherin and N-cadherin. Some lncRNAs regulate this process to promote or suppress the metastasis of melanoma. For example, CASC15 activates the Wnt–β-catenin pathway, resulting in promotion of EMT [110]. SLNCR1 is overexpressed in melanoma cells and acts as an oncogene. The SLNCR1–SOX5 axis promotes EMT and downregulation of SLNCR1, resulting in a decrease in SOX5 protein levels and suppression of tumourigenesis [111]. Through the induction of EMT, NEAT1 stimulates the invasion and migration of melanoma cells [112].

The entire process of metastasis is controlled by various regulators, including matrix metalloproteinases (MMPs). This family of endopeptidases proteolytically processes numerous growth factors, adhesion molecules, cytokines, and receptors, actions that help sustain homeostasis but also mediate metastasis [113]. GAS5 suppresses the ability of melanoma cells to migrate and invade via inhibition of MMP2 expression and its activity [114]. MALAT1 acts as a ceRNA for miR-22, a tumour suppressor in melanoma. It also leads to overexpression of MMP14 and downregulation of MMP2 and E-cadherin. As a result, MALAT1 stimulates the invasion and migration of melanoma cells [17].

The lncRNAs that stimulate metastasis are usually correlated with poor prognosis. TTN-AS1 promotes tumourigenesis and metastasis. It stimulates titin expression by stabilising titin mRNA, increasing activity of the titin promoter, and promoting titin accumulation in the cytoplasm. Overexpression of both TTN-AS1 and titin is correlated with poor prognosis of melanoma [115]. Similarly, LINC00518 stimulates melanoma metastasis and is correlated with a poor prognosis [116]. Table 1 summarises the involvement of lncRNAs in cell proliferation, apoptosis, and EMT.

### 4.4. Angiogenesis

Angiogenesis, the formation of new blood vessels from the pre-existing vasculature, is a hallmark of melanoma progression. It enables tumour expansion beyond 1–2 mm in thickness, nutrient supply, and metastatic dissemination. In melanoma, this process transitions from the radial to the vertical growth phase, marked by increased vascular density and leaky, tortuous vessels. Unlike physiological angiogenesis, tumour-associated neovascularisation involves dysregulated sprouting, intussusception, and vessel co-option, driven by pro-angiogenic factors secreted by melanoma cells, stromal components, and the hypoxic tumour microenvironment. The key mechanisms include vascular endothelial growth factor (VEGF) pathway activation, hypoxia-inducible factor (HIF)-mediated adaptation to hypoxia, and endothelial cell activation leading to vessel remodelling. These interconnected pathways not only sustain tumour growth but also contribute to therapeutic resistance, as evidenced by clinical trials and preclinical models [117,118].

VEGF-A is the master regulator of angiogenesis in melanoma. It promotes endothelial cell proliferation, migration, microvascular permeability, and differentiation of tip and stalk cells during capillary sprouting, all of which are essential to angiogenesis. VEGF-A binds primarily to vascular endothelial growth factor receptor 1 (VEGFR-1; Flt-1) and VEGFR-2 (KDR/Flk-1), initiating intracellular signalling cascades that regulate angiogenesis, while VEGF-C and VEGF-D preferentially bind VEGFR-3, promoting lymphangiogenesis and supporting metastatic dissemination through lymphatic channels. Alternative splicing of VEGFA results in multiple isoforms (VEGF121, VEGF165, and VEGF189), each with unique biochemical properties and tissue distributions. VEGF121, a freely soluble isoform, diffuses readily through the extracellular matrix, facilitating widespread angiogenic signalling, whereas VEGF189 binds strongly to heparin and extracellular matrix components, remaining largely localised. Intermediate isoforms, such as VEGF165, exhibit both soluble and matrix-bound characteristics. Importantly, anti-angiogenic VEGFXXXb isoforms, generated via distal splicing of exon 8, are frequently downregulated in aggressive melanomas, contributing to the so-called ‘angiogenic switch’ that favours tumour vascularisation [119,120].

VEGF expression is regulated at multiple levels. Transcriptionally, HIF-1α, stabilised under hypoxic conditions, induces VEGF-A expression. Post-transcriptional modulation occurs through CDK4 and CDK6, which influence VEGF-A transcription and secretion. Additionally, VEGF can be sequestered in the extracellular matrix, associated with heparan sulphate proteoglycans or basement membranes, and released upon proteolytic degradation by MMPs, amplifying local angiogenic signalling [121]. VEGF-A acts in both paracrine and autocrine fashions; in the latter case, melanoma cells expressing VEGFR-2 or neuropilins can respond directly to VEGF-A, supporting vascular mimicry and enhancing tumour progression. Experimental evidence has demonstrated the functional importance of VEGF-A isoforms in melanoma. In vivo studies in melanoma xenografts reveal that expression of soluble isoforms, such as VEGF121 and VEGF165, promotes rapid tumour growth and extensive angiogenesis, whereas matrix-bound VEGF189 is associated with limited vascularisation and tumour dormancy. The balance between pro-angiogenic and anti-angiogenic isoforms critically determines tumour aggressiveness and metastatic potential [120,122,123].

Hypoxia is a defining characteristic of solid tumours, including melanoma. As tumours expand, the distance between proliferating cells and existing capillaries increases, leading to regions of low oxygen tension. Hypoxia triggers stabilisation of HIF-1α, a transcription factor normally degraded under normoxic conditions via prolyl hydroxylation and recognition by the von Hippel-Lindau (VHL) E3 ubiquitin ligase complex. Stabilised HIF-1α translocates to the nucleus, dimerises with HIF-1β (ARNT), and binds hypoxia response elements (HREs) in the promoters of target genes, orchestrating a transcriptional programme that promotes angiogenesis, metabolic adaptation, invasion, and survival [122,124].

HIF-1α induces the transcription of key pro-angiogenic factors, including VEGF-A and basic fibroblast growth factor (bFGF), facilitating endothelial proliferation and vessel formation. It also regulates glucose transporters (GLUT1 and GLUT3) and glycolytic enzymes (hexokinase 1 [HK1], HK2, and lactate dehydrogenase A [LDHA]), enabling tumour cells to maintain energy production under low oxygen conditions through the Warburg effect, characterised by high rates of aerobic glycolysis and lactate production. Furthermore, HIF-1α modulates pyruvate dehydrogenase kinase 1 (PDK1), inhibiting the conversion of pyruvate to acetyl-CoA, thereby limiting mitochondrial respiration and reinforcing glycolytic metabolism. HIF-1α intersects with multiple oncogenic signalling pathways relevant to melanoma progression, including PI3K–Akt–mTOR, RAS–RAF–MEK–ERK, Janus kinase (JAK)–STAT, Wnt–β-catenin, Notch, and NF-κB [125,126,127]. These interactions amplify pro-angiogenic signalling, enhance tumour proliferation and motility, and contribute to resistance to apoptosis. Clinical studies consistently demonstrate that elevated HIF-1α expression correlates with increased VEGF expression, microvascular density, and poor prognosis in melanoma patients, underscoring its role as both a biomarker and therapeutic target [128,129].

The activation of endothelial cells in melanoma entails a transition from a dormant state to one of active division and relocation, driven by factors released from tumour cells [124,130]. VEGF-A initiates this by preparing endothelial cells for sprouting-based vessel growth, resulting in the emergence of tip cells equipped with filopodia that are directed by delta-like canonical Notch ligand 4 (DLL4)–Notch pathways. Following stalk cells then expand to form lumens. A notable feature in human melanoma metastases is intussusceptive angiogenesis, which divides existing vessels through the creation of internal pillars and depends on MMP9, along with the involvement of macrophages and T cells; such pillars appear infrequently in patient-derived xenografts (PDXs) or models involving BRAF/PTEN alterations, underscoring differences specific to humans [118,131].

Angiogenesis in melanoma is regulated not only by classical protein factors (VEGF-A, bFGF, PDGF, angiopoietins) but also by lncRNAs, which have emerged in recent years as critical modulators of the angiogenic switch and tumour vascularisation. Among the best-characterised pro-angiogenic lncRNAs in melanoma are MALAT1, HOTAIR, BANCR, SLNCR1, and DANCR. MALAT1 promotes VEGF-A secretion and endothelial cell proliferation/migration by sponging miR-140 and miR-145 and by activating the VEGF/VEGFR2 axis [132,133]. HOTAIR recruits the PRC2 complex, leading to epigenetic silencing of tumour-suppressor genes (e.g., PTEN) and subsequent upregulation of VEGF [134]. BANCR increases VEGF-C and VEGFR-3 levels, thereby strongly promoting lymphangiogenesis and nodal metastasis [12,135]. SLNCR1 enhances angiogenesis through epigenetic repression of SPRY2 via DNMT1 recruitment, whereas DANCR functions as a ceRNA by sequestering miR-5194, resulting in derepression of pro-angiogenic targets [136,137]. Conversely, certain lncRNAs exert anti-angiogenic effects. For example, CPS1-IT1 suppresses Cyr61, VEGF, and MMP-9 expression, inhibiting both angiogenesis and EMT, while NKILA blocks NF-κB signalling and reduces IL-8/VEGF secretion [138].

High expression of pro-angiogenic lncRNAs (MALAT1, BANCR, SLNCR1, SAMMSON) correlates with advanced clinical stage, the presence of metastases, and poorer overall survival, making them attractive prognostic biomarkers detectable in liquid biopsies [139]. Preclinical studies have demonstrated that silencing of MALAT1, HOTAIR, or SAMMSON using antisense oligonucleotides (ASOs) or gapmeRs dramatically reduces intratumoral vessel density and tumour growth and shows synergistic effects with BRAF/MEK inhibitors and bevacizumab. Thus, lncRNAs represent a novel layer of regulation within the angiogenic network of melanoma and offer promising dual-purpose therapeutic targets that simultaneously impair neovascularisation and directly kill cancer cells (Table 2).

### 4.5. Cancer Stem Cell Properties and Phenotypic Plasticity

Melanoma represents one of the most aggressive malignancies in humans, primarily due to its exceptional ability to dynamically alter its phenotype in response to intrinsic and extrinsic cues. This plasticity enables the persistence of melanoma-initiating cells (MICs), which exhibit stem-cell-like features and drive tumour heterogeneity, therapy resistance, and metastasis. MICs possess the capacity for self-renewal, differentiation, and dedifferentiation, sustaining continuous tumour propagation even under therapeutic pressure. Their maintenance is tightly regulated by epigenetic and transcriptional networks that orchestrate the expression of stemness-associated genes, including SOX2, OCT4, and NANOG [147,148]. The microenvironment exerts a pivotal influence on MIC survival. Oxygen levels, cytokines, growth factors, and therapeutic agents collectively shape the transcriptional state of melanoma cells, inducing reversible reprogramming events that support stemness and tumour persistence. Importantly, lncRNAs regulate these networks by modulating transcription, chromatin accessibility, and post-transcriptional gene expression to sustain the stem-like phenotype [149,150].

Stemness in melanoma is largely governed by a transcriptional triad—SOX2, OCT4, and NANOG—whose cooperative activity preserves pluripotent features and enhances tumourigenic potential. The expression of these factors is regulated by intricate epigenetic mechanisms as well as ncRNAs that act at both the transcriptional and post-transcriptional levels. For example, the lncRNA cardiac mesoderm enhancer-associated non-coding RNA (CARMN) modulates pluripotency by forming a complex with miR-143/mir-145, which in turn regulates the translation of SOX2, OCT4, NANOG, and SATB2. Within the nucleus, CARMN promotes chromatin remodelling at the SOX2 promoter, enhancing its transcriptional activation [150,151]. Other ncRNAs, including miR-873, have been implicated in stemness regulation through their influence on PD-L1 expression. Inhibition of miR-873 increases SOX2, OCT4, and NANOG levels, thereby enriching CD44^+^/CD24^−^ stem-like populations and enhancing self-renewal capacity [152]. These findings underscore the complex interplay between miRNAs, immune checkpoint molecules, and stemness maintenance in melanoma progression

Phenotype switching is one of the characteristic features of melanoma and considered a potential cause of treatment resistance. In general, melanoma tumours are divided into four main phenotypes, including melanocytic, transitory, neural-crest like, and undifferentiated [153]. Each subtype is associated with different sensitivity to systemic treatment. In case of molecular mechanisms, high expression of MITF and SRY-box transcription factor 10 (SOX10) with low expression of dedifferentiation marker AXL are characteristic of the invasive phenotype. Conversely, upregulation of AXL and downregulation of MITF and SOX10 are associated with the invasive phenotype [154]. The transition towards invasive or dedifferentiated phenotypes is often induced by stress signals such as hypoxia, nutrient deprivation, or exposure to targeted therapies (e.g., MAPK pathway inhibitors). During treatment, melanoma cells can adopt transient, drug-tolerant states, which precedes dedifferentiation into a neural crest stem–like cell state. These adaptive states confer survival advantages and contribute to both targeted therapy and immunotherapy resistance [34,155,156,157]. Dedifferentiation enables melanoma cells to revert to a more primitive, stem-like state, reactivating developmental programmes reminiscent of neural crest progenitors. This process is characterised by the loss of melanocytic markers (MITF, TYR, and MLANA) and the acquisition of mesenchymal and neural crest markers (AXL, ZEB1, NGFR). Although dedifferentiated melanoma cells exhibit reduced proliferation, they have a higher invasive and metastatic potential. Conversely, trans-differentiation allows melanoma cells to adopt alternative lineage identities without reverting to a pluripotent state, contributing to intratumoural heterogeneity and adaptive resilience [151,156,158].

Researchers study molecular and structural events trying to comprehensively understand phenotypes leading to drug resistance. In a recent study by Simiczyjew et al., the authors demonstrated that cells resistant to BRAF and MEK inhibitors undergo cytoskeletal rearrangement that is linked to YAP nuclear localisation. These changes contribute to cell adhesion, invasion, and spreading [159]. Unsurprisingly, researchers are evaluating the potential to target cells of the invasive phenotype to suppress mechanisms leading to treatment resistance [160].

**Figure 5:**
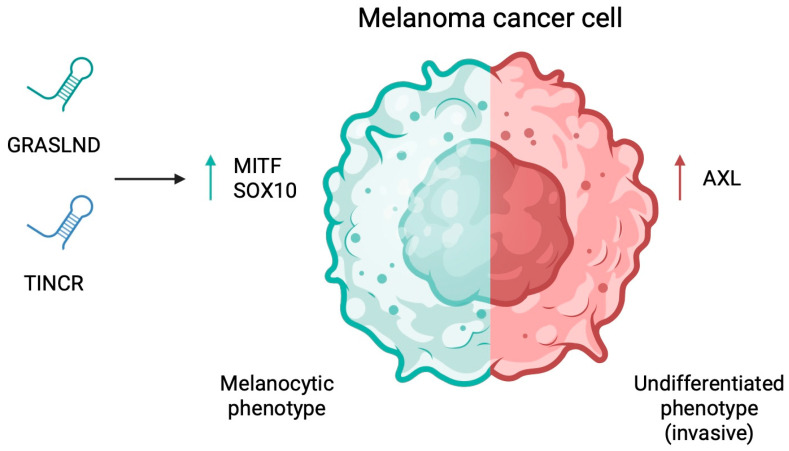
Melanoma cells can induce phenotype switching that promotes treatment resistance. Long non-coding RNA are involved in this process by regulating the expression of typical phenotype markers. Relationships between GRASLND and TINCR with MITF and SOX10 highlight their involvement in phenotype determination. Created in BioRender. Physiology, D. (2026) https://BioRender.com/hfy1mw8.

lncRNAs are involved in the regulation of melanoma phenotype switching. The lncRNA GRASLND is upregulated in differentiated cells relative to mesenchymal undifferentiated melanoma cells. Mechanistically, it showed positive correlation with MITF and melanocytic marker MelanA. GRASLND knockdown promoted the non-proliferative state of melanoma cells [161]. TINCR is another lncRNA demonstrated to be involved in the proliferative phenotype. Specifically, its silencing promoted the invasive cancer phenotype. Secondly, an association with MITF was demonstrated, with depletion of the latter causing reduced TINCR levels [21] (Figure 5).

### 4.6. Metabolic Reprogramming

In addition to genetic drivers, melanoma progression and therapeutic resistance also depend on the cell’s ability to dynamically remodel its metabolic network. Tumour cells continuously adjust energy production, biosynthetic pathways, and redox homeostasis to sustain proliferation in a fluctuating microenvironment defined by hypoxia, nutrient competition, and therapeutic stress. Within this landscape, three axes of metabolic adaptation emerge as particularly relevant to melanoma biology: the SAMMSON-mediated regulation of mitochondrial metabolism, the oncogenic rewiring of glucose and glutamine utilisation, and the modulation of lipid metabolism within both tumour and immune compartments. Together, these interconnected processes construct a flexible metabolic architecture that allows melanoma to survive targeted therapy and immune surveillance [162,163].

SAMMSON represents one of the most distinctive examples of a non-coding regulator that orchestrates mitochondrial function in melanoma. Co-amplified with MITF and transcriptionally linked to the melanocytic lineage programme, SAMMSON sustains the bioenergetic and biosynthetic capacity of tumour cells by directly coordinating mitochondrial translation and integrity. It interacts with the mitochondrial protein p32, a multifunctional chaperone involved in ribosomal RNA processing and mitochondrial ribosome assembly. Through this interaction, SAMMSON facilitates maturation of the 16S rRNA component of the large mitoribosomal subunit, thereby enhancing the synthesis of mitochondrial-encoded respiratory-chain proteins and supporting efficient oxidative phosphorylation [145]. Loss of SAMMSON leads to profound mitochondrial dysfunction. Electron microscopy analyses have revealed disrupted cristae, while metabolic profiling has demonstrated a decline in oxygen-consumption rates and ATP production. This bioenergetic collapse provokes compensatory activation of glycolysis, but this shift is insufficient to maintain cell viability, leading to intrinsic apoptosis mediated by cytochrome *c* release. Thus, melanoma cells exhibit a strong dependence on SAMMSON for maintaining mitochondrial translation fidelity and energy output. SAMMSON also exerts control beyond the organelle. By binding 5′-3′ exoribonuclease 2 (XRN2), it modulates cytoplasmic RNA stability and translation, coupling cytosolic and mitochondrial protein synthesis. This coordination ensures a balanced stoichiometry between nuclear-encoded and mitochondrially encoded components of the oxidative phosphorylation machinery, preventing proteotoxic stress within mitochondria. Such coupling becomes critical under metabolic or therapeutic stress, when melanoma cells rely on synchronised translational programmes to restore homeostasis [139,144]. In addition to sustaining bioenergetics, SAMMSON contributes to metabolic plasticity by influencing mitochondrial dynamics and signalling. Enhanced SAMMSON expression promotes mitochondrial fusion and suppresses fission, yielding an elongated network associated with higher respiratory efficiency. It also stabilises peroxisome proliferator-activated receptor gamma coactivator 1-alpha (PGC-1α), the master regulator of mitochondrial biogenesis, reinforcing a feed-forward circuit that expands mitochondrial mass and antioxidant capacity. This mitochondrial reinforcement provides a survival advantage against oxidative damage induced by targeted inhibitors or immune effector mechanisms. Taken together, SAMMSON is a pivotal metabolic oncogene that links transcriptional lineage identity to mitochondrial robustness. Hence, it could be a useful therapeutic target to disable the energetic core of melanoma [144,164,165].

While mitochondrial metabolism provides the foundation for oxidative energy production, melanoma cells simultaneously exploit glycolysis and glutaminolysis to meet biosynthetic and redox demands. Oncogenic activation of the MAPK pathway through BRAF mutations drives this reprogramming by upregulating glucose transporters and glycolytic enzymes via MYC and HIF-1α. The resulting ‘glycolytic phenotype’ supports rapid ATP generation and supplies intermediates for nucleotide, amino acid, and lipid synthesis. However, melanoma displays a hybrid metabolic state rather than a purely glycolytic one. Depending on the environmental cues, cells toggle between glycolysis and oxidative phosphorylation, and this flexibility underlies resistance to BRAF and MEK inhibitors [140,165]. A key mediator of this adaptive equilibrium is the transcription factor TFEB, traditionally recognised as the master regulator of lysosomal biogenesis and autophagy. Under basal conditions TFEB remains cytoplasmic and phosphorylated by ERK, but upon inhibition of the MAPK pathway or nutrient deprivation it translocates to the nucleus, activating genes involved in autophagy, lipid catabolism, and mitochondrial biogenesis. Through these programmes, TFEB restores metabolic balance when glycolytic flux is suppressed. TFEB activation increases mitochondrial mass, enhances oxidative phosphorylation, and stimulates the synthesis of cholesterol and phospholipids required for membrane remodelling. At the same time, it reduces excessive lactate production, alleviating acidosis within the tumour microenvironment [146]. TFEB-driven autophagy functions as an emergency recycling system that liberates amino acids and lipids from intracellular stores.

Glutamine, the most abundant amino acid in circulation, becomes a critical substrate feeding the tricarboxylic-acid (TCA) cycle through conversion to α-ketoglutarate. Under BRAF inhibition, melanoma cells upregulate glutaminase and transaminases to maintain anaplerosis, enabling survival despite reduced glycolytic input. This metabolic rerouting depends partly on TFEB-mediated transcriptional control of lysosomal enzymes that supply glutamine from protein degradation. Beyond energy maintenance, glucose and glutamine metabolism contribute to redox regulation. NADPH produced through the pentose-phosphate pathway and malic enzyme reactions counterbalances the reactive oxygen species generated by mitochondrial respiration. TFEB indirectly supports this antioxidant network by maintaining mitochondrial quality control through mitophagy. The coordination between glycolytic, glutaminolytic, and oxidative pathways allows melanoma cells to withstand metabolic stress, sustain proliferation, and resist apoptosis induced by targeted therapy [162,165]. At the systems level, these regulatory loops create dynamic feedback between oncogenic signalling and metabolism. When BRAF–ERK activity is high, cells favour glycolysis and biosynthetic growth; when the pathway is pharmacologically suppressed, TFEB-driven mitochondrial and autophagic programmes take over, restoring ATP and redox balance. This bidirectional communication exemplifies the adaptive resilience of melanoma and explains the transient efficacy of MAPK pathway inhibitors. Disrupting this metabolic compensation—by targeting TFEB activation, glutamine metabolism, or mitochondrial biogenesis—may help prolong therapeutic responses and reduce the risk of relapse [140].

Among the most versatile adaptations of melanoma cells is their ability to reprogramme lipid metabolism. Lipids serve as structural components of membranes, energy reservoirs, and signalling molecules that influence cell survival, inflammation, and immune recognition. Melanoma exhibits a pronounced capacity to synthesise, store, and oxidise lipids depending on microenvironmental conditions [166]. De novo lipogenesis is enhanced through activation of sterol-regulatory element–binding proteins (SREBPs) downstream of PI3K–AKT and MAPK signalling. This drives expression of fatty-acid synthase, acetyl-CoA carboxylase, and stearoyl-CoA desaturase, leading to accumulation of monounsaturated fatty acids that maintain membrane fluidity and protect against lipid peroxidation. Concurrently, TFEB and other members of the MiT/TFE family stimulate genes involved in fatty-acid uptake and β-oxidation, endowing melanoma cells with the ability to switch between lipid synthesis and oxidation. Such a dual capacity enables survival under nutrient limitation: cells can catabolise stored triglycerides in lipid droplets via autophagic lipolysis to fuel mitochondrial respiration.

This metabolic versatility extends beyond tumour-intrinsic functions and profoundly affects the tumour microenvironment. The accumulation of lipids within melanoma cells alters the composition of extracellular vesicles and secreted metabolites, which in turn influence infiltrating immune cells. Lipid-enriched exosomes can deliver unsaturated fatty acids and oxidised phospholipids to macrophages and T cells, reprogramming their metabolism towards an immunosuppressive phenotype. In CTLs, chronic exposure to lipid-rich conditions induces metabolic exhaustion characterised by excessive lipid droplet formation, mitochondrial depolarisation, and diminished cytokine production [167]. Mechanistically, this immune dysfunction is linked to the MAPK–STAT3–cytosolic phospholipase A2α (cPLA2α) axis. Activation of cPLA2α promotes the release of arachidonic acid from membrane phospholipids, fuelling prostaglandin synthesis and suppressing effector T cell function. In senescent or chronically stimulated T cells, upregulation of this pathway enhances lipid remodelling and perpetuates oxidative stress, leading to irreversible metabolic paralysis. Thus, the tumour’s lipid landscape acts as both a nutrient source and an immunomodulatory signal [168,169].

Therapeutically, lipid metabolism represents an attractive but complex target. Inhibiting fatty-acid synthase or SREBP signalling can limit melanoma growth but may trigger compensatory activation of lipid uptake and oxidation pathways. Conversely, blocking carnitine palmitoyltransferase I (CPT1A)-dependent β-oxidation sensitises tumour cells to oxidative stress, although it may also impair T cell endurance, because effector memory T cells rely on fatty acid oxidation for persistence. Therefore, rational combination therapies require precise temporal and cell-type-specific modulation of lipid pathways to suppress tumour metabolism while preserving antitumour immunity [166,170].

Recent studies suggest that lipid metabolism intersects with ferroptosis sensitivity in melanoma. High expression of desaturases and glutathione peroxidase 4 confers resistance to lipid-peroxidation-induced cell death. Targeting this protective axis may synergise with immune checkpoint blockade by enhancing tumour immunogenicity through oxidative lipid damage. Understanding the cross-talk between tumour lipid metabolism and immune function will be essential for designing strategies that convert metabolic vulnerability into therapeutic advantage [163].

Taken together, metabolic reprogramming in melanoma is not a collection of isolated phenomena; rather, it is an integrated adaptive system linking mitochondrial function, nutrient utilisation, and lipid homeostasis to oncogenic signalling and immune evasion. SAMMSON ensures mitochondrial translation and structural integrity, sustaining the energetic backbone of the tumour. TFEB and associated transcriptional networks provide a flexible interface between glycolytic and oxidative states, ensuring survival under therapeutic stress. Lipid metabolic rewiring expands this adaptability, enabling both tumour resilience and suppression of immune surveillance.

## 5. Clinical Value of Long Con-Coding RNA in Melanoma

Current evidence highlights a key role of lncRNA in the pathophysiology of melanoma. Regulation of signalling pathways, angiogenesis, differentiation status, and metabolic reprogramming are elements deeply involved in oncogenesis. LncRNAs were demonstrated to regulate these processes, thus mediating melanoma progression. Along with studies researching lncRNA-related pathways, clinical analyses are also being performed. These evaluate if lncRNA could become predictive and prognostic factors, as well as diagnostic biomarkers. Such parameters are strongly needed in modern oncology, as accumulating biomarkers are being used to administer adequate systemic treatment. For instance, EGFR inhibitors are not administered in colorectal cancer (CRC) patients harbouring NRAS or KRAS mutations. Moreover, CRC patients with high microsatellite instability can be treated with immunotherapy. In melanoma, combinations of BRAF and MEK inhibitors are recommended in patients with BRAF-mutated tumours [3]. Hypothetically, lncRNAs could also serve as parameters that will guide therapeutic decisions. Circulating lncRNAs can be detected in the blood and could be utilised in liquid biopsies. LINC00173 was evaluated as a potential diagnostic and prognostic biomarker. Its tissue expression showed promising diagnostic value, with area under curve (AUC) in the ROC analysis of 0.7695. Additionally, its high expression was correlated with worse overall survival, advanced stage, and lymph node metastasis [171]. The diagnostic and prognostic potential of several lncRNAs were confirmed in a meta-analysis [172]. Furthermore, lncRNAs were predicted as potential predictive biomarkers to immunotherapy. In a bioinformatics study, Lu et al. [173] analysed 62 lncRNAs associated with 17 immunological gene sets. In the survival analysis, the authors demonstrated that a few lncRNAs can distinguish responders and non-responders, which translated into a significant overall survival difference. A prospective clinical evaluation is warranted to confirm these predictions. Additionally, Oliver et al. identified over 100 differently expressed lncRNAs between responders and non-responders to immunotherapy in patients with metastatic melanoma [174]. When looking at lncRNAs individually, it can be observed that these molecules mediate the immune status of tumour tissues. This is consistent with our knowledge that the immunotherapy response is greatly dependent on the composition of the tumour microenvironment. When comparing colon cancer and melanoma, Munteanu et al. [175] found that colon cancer tissue has a greater expression of FENDRR, the tumour suppressor mentioned previously in this article. The expression of lncRNA was associated with a greater presence of pro-inflammatory cytokines in colon cancer. Immune cell composition differed as well, with greater presence of CD3+CD4+ cells in the colon tissue. In another analysis by Toker and colleagues [176], the researchers observed significant upregulation of NEAT1 in the tissue of melanoma patients that responded to immunotherapy. Gene set enrichment analysis further demonstrated that NEAT1 promotes immune responses, with interferon signalling being the most enriched set. An interesting pathway was recently published by Cinque et al. [177]. The authors demonstrated that the lncRNA LISRR interacts with ribosomes to enhance the translation of immunosuppressive proteins. As a result, the molecule can mediate immunotherapy response.

## 6. Limitations and Future Areas

**Figure 6:**
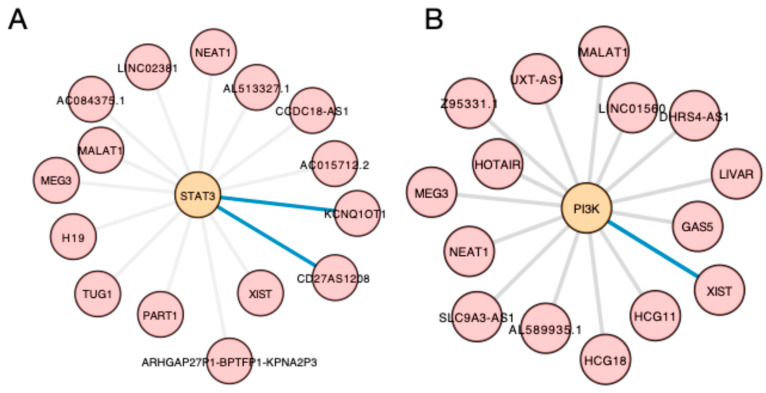
Predicted and experimentally validated representative interaction networks of lncRNA and (**A**) STAT3 and (**B**) PI3K in melanoma. Apart from including lncRNA molecules with direct or indirect (ceRNA) pathway targets, representative samples from hundreds of identified molecules by NCpath were included. Interactions deeper than ceRNA, like those demonstrated in the case of HOTAIR, were not highlighted [180]. The figure demonstrates that even with known interactions between lncRNAs and signalling cascades observed in different disease models, such experiments were not yet performed in melanoma. Furthermore, the figure also demonstrates molecules without proper annotation. Orange circles represent target proteins. Simplification was performed in case of PI3K, as it is a family of enzymes with different molecules targeting different enzymes. Blue lines indicate known and validated interactions discussed in this review. Grey lines show interactions not experimentally demonstrated in melanoma.

Accumulating studies continue to provide insight into the involvement of lncRNAs in oncogenesis. With numerous identified lncRNAs so far and the complexity of interactions that these molecules are involved in, the current research area seems very broad. With this being true, there is still enormous gap to fill to understand fully the wide functions induced by lncRNAs. To demonstrate the small area we know so far, we have utilised bioinformatics tools to dig into signalling pathways. Using the NCPatch tool (available at http://ncpath.pianlab.cn/#/Home) [178], we have searched the JAK/STAT and PI3K/Akt signalling pathways and extracted the experimental and predicted lncRNAs that interacted with these pathways. Within these cascades, we focused on STAT3 and PI3K as the elements that we discuss in this article. Subsequently, we have constructed small exemplary interaction networks using Cytoscape (version 3.10.3) [179]. The networks were constructed to include the lncRNAs discussed in this manuscript, together with widely known and recognised lncRNAs form the database, as well as unbiased selection of molecules. Subsequently, a literature review was performed to check if lncRNA–STAT or –PI3K interactions were experimentally validated in melanoma (Figure 6). With these small examples of networks, it is demonstrated how little is known about the interactions of lncRNAs with major oncogenic signalling pathways.

Importantly, certain limitations for ncRNA-based studies need to be addressed. With the previously mentioned abundance of molecules and interactions, only experimentally validated molecules should proceed to clinical or pharmacological evaluation. Apart from predicted data, experiments using clinical samples frequently use small sample sizes. Furthermore, some lncRNAs are shown to promote or suppress oncogenesis depending on disease model cellular context. These differences will need to be addressed as well.

## 7. Conclusions

Researchers continue to explore the comprehensive roles of lncRNAs in the pathophysiology of melanoma. The current evidence highlights their involvement in regulating the signalling pathways, proliferation, apoptosis, and metabolism of cancer cells. These findings suggest that lncRNAs could also be responsible for mediating the response to therapy. Considering the immunoregulatory properties of lncRNAs, more research is required to determine their role in the response to immunotherapy. This area is especially relevant in light of the current wide use of this form of treatment in patients with melanoma. Similarly to other types of ncRNAs, lncRNAs are involved in large regulatory networks. Sometimes these molecules appear to have contradictory functions. These results could result from different variants of lncRNAs, technical aspects (cell/animal model used), and/or the cellular context. As demonstrated in this review, lncRNAs are implicated in feedback loops and they have many upstream regulators. As they are important regulators of gene expression, the monitoring of lncRNAs could become a promising method to evaluate melanoma pathogenesis and treatment response. Hypothetically, the expression of these molecules could be manipulated to increase the probability of responding to treatment.

## Figures and Tables

**Table 1:** The involvement of lncRNAs in cell proliferation, apoptosis, and epithelial-to-mesenchymal transition in melanoma.

LncRNA	Expression	Action	Effect	Reference
BANCR	up	microRNA-204 down-regulator (Notch2 pathway)	Promotion of EMT and tumorigenesis	[94]
NEAT1	up	miR-495-3p inhibitor and E2F3 inductor	Promotion of EMT and tumorigenesis	[95,112]
SNHG5	up	miR-26a-5p inhibitor	Biomarker of advanced stage of melanoma	[98]
SPRY4-IT1	up	miR-22-3p up-regulator	Melanoma cell proliferation, invasion, migration, and EMT stimulator	[42]
GAS6-AS2	up	GAS6 expression up-regulator	Melanoma cell proliferation stimulator, biomarker of advanced stage and poor prognosis	[100]
MALAT1	up	CyclinD1 and CyclinE1 expression stimulator	Melanoma cell proliferation stimulator; apoptosis inhibitor	[17,101]
FOXC2-AS1	up	silencing p15	Melanoma cell proliferation stimulator; marker of poor prognosis	[9]
lncRNA-ATB	up	miR-590-5p sponge	Melanoma cell proliferation, invasion, and migration stimulator	[102]
XIST	up	miR-21 sponge		[39]
BASP1-AS1	up	Notch pathway activator	Oncogene expression stimulator	[103]
MIAT	up	PI3K/AKT pathway activator c-MYC and cyclin D1 expression stimulator		[40]
lncRNA-TTN-AS1	up	Cyclin D1, CDK2, and CDK4 up-regulator; titin expression stimulator	Apoptosis inhibitor; marker of poor prognosis; EMT stimulator	[115]
FENDRR	down	Cyclin E1, Cyclin D1, CDK4, and CDK6 down-regulator	Melanoma cell growth suppressor	[90]
TRMP	up	p27 inhibitor	Metastasis promotor	[104]
TRMP-S	up	p27 inhibitor	Melanoma cell proliferation stimulator, cell senescence suppressor	[105]
LINC-PINT	down	Recruits EZH2, leading to H3K27 trimethylation	Tumorigenesis and cell migration suppressor	[10]
GAS5	down	Bcl-2 expression stimulator	Melanoma cell apoptosis	[57,114]
LHFPL3-AS1	up	Bcl-2 degradation inhibitor	Apoptosis suppressor	[20]
CASC15		Wnt/β-catenin pathway activator	Melanoma cell proliferation and EMT stimulator	[110]
SLNCR1	up	SLNCR1/SOX5 axis	EMT stimulator	[111]

**Table 2:** Potential mechanisms of long non-coding RNAs regulating angiogenesis in melanoma.

lncRNA	Mechanism	Effect	References
MALAT1	Sponges miR-140/miR-145/miR-497 → ↑ VEGF-A, FGF2; stabilises HIF-1α; activates VEGFR2	Strong promotion of angiogenesis and vessel maturation	[135,138,140]
HOTAIR	Recruits EZH2/PRC2 → epigenetic silencing of PTEN, P21 → ↑ PI3K/AKT → ↑ HIF-1α/VEGF	Increased VEGF secretion even under normoxia	[134,140,141,142]
BANCR	Activates NF-κB → ↑ VEGF-C and VEGFR-3	Strongly promotes lymphangiogenesis and nodal metastasis	[140,142]
SLNCR1	Scaffolds AR/Brn3a at MMP9 promoter + recruits DNMT1 to silence SPRY2	Enhances invasion and angiogenesis	[12,140]
DANCR	ceRNA for miR-5194 → derepresses multiple pro-angiogenic targets	Increased tube formation and endothelial recruitment	[136]
SAMMSON	Interacts with p32/CARF in mitochondria → maintains OXPHOS under hypoxia → ↑ VEGF	Hypoxia-driven angiogenesis; high vessel density in PDX models	[139,140,143,144,145,146]
CPS1-IT1	Inhibits Cyr61, VEGF, MMP-9 expression; blocks ERK signalling	Strong suppression of angiogenesis and EMT	[86]
NKILA	Directly binds and inhibits IκB phosphorylation → blocks NF-κB → ↓ IL-8/VEGF	Reduced inflammatory cytokine-driven angiogenesis	[85]
MEG3	Stabilises p53 → ↓ VEGF; competes with miR-183 for binding	Decreased angiogenesis and tumour growth	[140,142]
GAS5	Sponges miR-210 → inhibits HIF-1α stabilisation under hypoxia	Reduced VEGF secretion and vessel formation	[140,142]

↓ – downregulation/decreased activity; ↑ - upregulation/ increased activity

## Data Availability

Not applicable.

## Abbreviations

The following abbreviations are used in this manuscript:

ncRNA	Non-coding RNA
lncRNA	Long non-coding RNA
miRNA	microRNA
UVR	Ultraviolet radiation
EZH2	Enhancer of zeste homolog 2
PRC2	Polycomb repressive complex 2
eRNA	Enhancer RNA
ceRNA	Competing endogenous RNA
ATF4	Activating transcription factor 4
uORF	Upstream open reading frame
eIF2α	Eukaryotic initiation factor 2 α subunit
EEF2	Elongation factor 2
BANCR	BRAF-activated non-protein coding RNA
HDAC	Histone deacetylase
PD-1	Programmed cell death protein 1
CDK	Cyclin-dependent kinase
CDKN1C	Cyclin-dependent kinase inhibitor 1C
MEG3	Maternally expressed gene 3
EMT	Epithelial-to-mesenchymal transition
FOXP3	Forkhead box P3
MDM2	Mouse double minute 2 homolog
HGF	Hepatocyte growth factor
TRMP	TP53-regulated modulator of p27
